# Effects of COVID-19 Pandemic in Patients with a Previous Phrenic Nerve Transfer for a Traumatic Brachial Plexus Palsy

**DOI:** 10.1055/s-0044-1787296

**Published:** 2024-06-12

**Authors:** Mariano Socolovsky, Johnny Chuieng-Yi Lu, Francisco Zarra, Chen Kuan Wei, Tommy Nai-Jen Chang, David Chwei-Chin Chuang

**Affiliations:** 1 Division of Neurosurgery, Hospital de Clínicas, University of Buenos Aires School of Medicine, Buenos Aires, Argentina; 2 Division of Reconstructive Microsurgery, Department of Plastic and Reconstructive Surgery, Chang Gung Memorial Hospital, Chang Gung University, Taoyuan, Taiwan; 3 Department of Neurosurgery, Sanatorio Guemes, University of Buenos Aires School of Medicine, Buenos Aires, Argentina

**Keywords:** brachial plexus injury, phrenic nerve transfer, COVID-19 acute infection, respiratory symptoms

## Abstract

**Background** With the advent of the coronavirus disease 2019 (COVID-19) pandemic, some doubts have been raised regarding the potential respiratory problems that patients who previously underwent a phrenic nerve transfer could have.

**Objectives** To analyze the effects of the coronavirus infection on two populations, one from Argentina and another from Taiwan. Specific objectives were: (1) to identify the rate of COVID in patients with a history of phrenic nerve transfer for treatment of palsy; (2) to identify the overall symptom profile; (3) to compare Argentinian versus Taiwanese populations; and (4) to determine if any phrenic nerve transfer patients are at particular risk of more severe COVID.

**Methods** A telephonic survey that included data regarding the number of episodes of acute COVID-19 infection, the symptoms it caused, the presence or absence of potential or life-threatening complications, and the status of COVID-19 vaccination were studied. Intergroup comparisons were conducted using the nonparametric Mann–Whitney U test, with categorical variables conducted using either the Pearson χ2 analysis or the Fisher's exact test, as appropriate.

**Results** A total of 77 patients completed the survey, 40 from Taiwan and 37 from Argentina. Fifty-five (71.4%) developed a diagnosis of COVID. However, among these, only four had any level of dyspnea reported (4/55 = 7.3%), all mild. There were also no admissions to hospital or an intensive care unit, no intubations, and no deaths. All 55 patients isolated themselves at home.

**Conclusions** It can be concluded that an acute COVID-19 infection was very well tolerated in our patients. (Level of evidence 3b, case reports).

## Introduction

The phrenic nerve as donor for nerve reconstruction in traumatic brachial plexus injuries (BPI) is commonly used in several specialized centers worldwide. 1 2 3 4 5 6 7 8 9 10 11 12 13 It is a convenient donor that can be easily harvested at the supraclavicular region during brachial plexus exploration and can reach important target nerves, such as the branches of the upper trunk with nerve transfer, or to the musculocutaneous nerve via nerve graft. 14 Including the phrenic nerve in the arsenal of donor nerves could be essential in brachial plexus reconstruction, especially in severe BPI like panplexus or C5–C8 injuries when the scarcity of donors renders reconstruction of multiple functions nearly impossible. Contraindications include obese patients, age < 3 years old, previous thoracic trauma, and arguably those with chronic lung disease and old age. It has been reported that the respiratory function meliorates after this nerve transfer in a certain period of time. 10 11 12 13 14

However, the coronavirus disease 2019 (COVID-19) pandemic has arisen in the world, and multiple studies have found permanent respiratory deficits after severe bouts of pneumonia 15 16 17 18 19 20 21 22 23 24 and also directly affecting the phrenic nerve, producing its palsy, thus potentially exacerbating the respiratory insufficiency in those cases where the phrenic nerve was used as the donor. 7 18 25

To analyze the effects that the infection by coronavirus might have produced on them, we studied two populations of different ethnicities who, prior to March 2020, underwent a phrenic nerve transfer to restore a lost function during a traumatic BPI. Two experienced nerve centers localized in different countries—Taiwan and Argentina—were enrolled. The reason for including these two countries was to analyze the results of two experienced centers that frequently use the phrenic nerve as a donor in BPIs, studying different ethnicities, geographies, and morphology of the COVID-19 pandemic. The objectives of the present work were: (1) to identify the rate of COVID in patients with a history of phrenic nerve transfer for treatment of palsy; (2) to identify the overall symptom profile and rate of significant respiratory symptoms in patients after phrenic nerve transfer who develop COVID-19; (3) to compare the Argentinian versus the Taiwanese patients; and (4) to compare COVID cases who report significant respiratory symptoms (other than cough) versus those who do not, to determine if any phrenic nerve transfer patients are at particular risk of more severe COVID. The rationale of this study was to answer an important question: did the previous medical history of complete sectioning of the phrenic nerve—and its potential negative effect on the respiratory function—really affect the course of the acute phase of a COVID-19 infection?

## Materials and Methods

### Materials

The total of patients who underwent phrenic nerve transfer to treat brachial plexus palsy at the two participating centers was included. Inclusion criteria were BPI patients who were operated before the COVID-19 pandemic and underwent a complete phrenic nerve section and suture to a distal nerve target to reinnervate one or more paralyzed muscles. Exclusion criteria for phrenic nerve harvesting were the same in both countries: age less than 3 years old or more than 50, obesity, chronic lung diseases such as chronic obstructive pulmonary disease or interstitial lung disease. A telephonic survey that included data regarding the number of episodes of acute COVID-19 infection, the symptoms it caused, the presence or not of potential or life-threatening complications, and the status of COVID-19 vaccination at the moment of the acute infections was studied ( Table 1 ). Exclusion criteria for being considered to be part of the present series are patients who were operated on after the COVID-19 pandemic, who were lost to follow-up, or who have a history of chronic lung diseases. The study was performed in full accordance with the Declaration of Helsinki II and both institution's ethics committees.

**Table 1:** Telephonic survey

1- Do you have any medical comorbidity?
2- Did you have COVID-19?
3- What number of episodes?
4- How was diagnosticated?
5- Which were the symptoms in each episode?
6- Had you ever been admitted to the hospital during the acute phase of the COVID/19 infection? In which episode/episodes? In which part of the hospital were you admitted? (ICU, normal room)
7- Were you intubated during any of the episodes?
8- Had you presented dyspnea during any acute episode of COVID-19?
9- Did you receive any vaccine for COVID-19? When and how many?

Abbreviations: COVID-19, coronavirus disease 2019; ICU, intensive care unit.

### Statistical Analysis

All continuous variables are expressed as means with ranges, with categorical variables summarized as absolute numbers and percentages. Since all the continuous variables (e.g., patient age) were found to have non-normal distributions using the Shapiro–Wilk test, intergroup comparisons were conducted using the nonparametric Mann–Whitney U test, with intergroup comparisons of categorical variables conducted using either the Pearson χ2 analysis or the Fisher's exact test, as appropriate. Since we considered the study exploratory, a two-tailed, unadjusted *p* ≤ 0.05 was used as the criterion for statistical significance. Given the minimal number of COVID-19 patients with respiratory symptoms beyond cough ( *N* = 4), multivariable analysis to identify predictors of respiratory dysfunction was considered inappropriate. SPSS version 28.0 (IBM Corp., Armonk, New York, United States) was the statistical software package used for all analyses.

## Results

A total of 77 patients or their families of a total of 168 completed the survey, 40 from Taiwan and 37 from Argentina. The earliest operative date included in this cohort was in January 2005, and the latest in February 2020, all before the beginning of the COVID-19 pandemic. Patients referred to have undergone the acute/s infection in a period ranging from March 2020 to January 2023, at the latest. As seen in the characteristics of the sample ( Table 2 ), patients were relatively young: almost 80% were under 40 years old, and predominantly (84.4%) were males, with Argentina and Taiwan reasonably equally represented. Of the 77 patients, 55 (71.4%) developed a diagnosis of COVID. However, among these, only four had any level of dyspnea reported (4/55 = 7.3%), all mild. There also were no admissions to hospital or an intensive care unit (ICU), no intubations, and no deaths. All 55 patients isolated themselves at home.

**Table 2:** Characteristics of the sample

Characteristics	*N*	%
Total number, *N*	77	
Age, (y)		
Mean (SD)	29.5 (11.6)	
Range	10–63	
Age group		
Under 20 y old	15	19.5
20–39 y old	46	59.7
40 y and over	16	20.8
Sex		
Females	12	15.6
Males	65	84.4
Country		
Argentina	37	48.1
Taiwan	40	51.9
Years (surgery to pandemic)		
<5	56	72.7
5–9	16	20.8
10+	5	6.5
Mean (SD)	3.71 (2.91)	
Range	0–15	
Diabetes	3	3.9
Hypertension (requiring treatment)	2	2.6
Any currently active comorbid condition	6	7.8
COVID		
Confirmed by nasal swab	41	53.2
Suspected due to close contact	14	18.2
Total number of COVID cases	55	71.4
Argentina ( *N* = 37 surgery patients)	25	67.6 a
Taiwan ( *N* = 40 surgery patients)	30	75.0 b
Swab test +		
Argentina ( *N* = 25 COVID cases)	11	44.0 c
Taiwan ( *N* = 30 COVID cases)	30	100.0 d
Symptoms ( *N* = 55 cases)		
Asymptomatic	9	16.4
Nonrespiratory symptoms	9	16.4
Cough but no dyspnea	33	60.0
Mild dyspnea	4	7.3
More severe dyspnea	0	0.0
Admitted to a hospital	0	0.0
Admitted to an ICU	0	0.0
Intubated	0	0.0
Died from COVID or any other cause	0	0.0

Abbreviations: COVID-19, coronavirus disease 2019; ICU, intensive care unit; SD, standard deviation.

a Out of 37 Argentinian patients.

b Out of 40 Taiwanese patients.

c Out of 27 Argentinian patients with COVID.

d Out of 30 Taiwanese patients with COVID.

In Table 3 , note that Taiwanese and Argentinian patients were quite different in several measures, with Taiwanese patients being younger, more likely to be female, almost all having developed COVID after at least one coronavirus vaccination (96.7 vs. 60.0%), and all having had COVID confirmed by nasal swab test (vs. just 44.4% among Argentinian patients). They also had undergone much more recent phrenic nerve transfers. Despite these differences, the two countries did not differ in the rate of COVID, number of episodes, or percentage of patients with respiratory symptoms (10% or less in both countries).

**Table 3:** Taiwanese versus Argentinian sample

Characteristics	Argentina	Taiwan	Test statistic (df)	Significance, *p*	Odds ratio (95% CI)
*N*	37	40			
Age, mean	32.9	26.40	MWU = 341.5 (1)	*p* < 0.001	NA
Range	15–51	10–63			NA
% under 20 y old	2.7%	35.0%	χ2 = 12.95 (2)	*p* = 0.002	NA
% 20–39 y old	70.3%	50.0%			
% 40 y and over	37.0%	15.0%			
% Female	5.4%	15.0%	χ2 = 5.61 (1)	*p* = 0.018	OR = 5.85 (1.83, 28.57)
Diabetes	0.0%	7.5%	FE	*p* = 0.24	NA
Hypertension (requiring treatment)	0.0%	5.0%	FE	*p* = 0.49	NA
Any currently active comorbid condition	2.7%	12.5%	χ2 = 2.57 (1)	*p* = 0.11	OR = 0.19 (0.02, 1.75)
% 5–9 y	2.5%	40.5%			
% ≥ 10 y	0.0%	13.5%			
Diagnosed with COVID-19, yes	67.6%	75.0%	χ2 = 0.52 (1)	*p* = 0.47	OR = 1.44 (0.53, 3.89)
When COVID diagnosed, before COVID vaccination	40.0%	3.3%	χ2 = 11.46 (1)	*p* < 0.001	OR = 19.23 (2.26, 165.7)
After COVID vaccination	60.0%	96.7%			
COVID confirmed by nasal swab	44.0%	100.0%	χ2 = 22.54 (1)	*p* < 0.001	OR = 3.73 (2.25, 6.18)
Number of episodes, mean	0.78	0.88	MWU = 810.0	*p* = 0.41	NA
Range	0–3	0–2			
0 episodes	32.4%	25.0%	χ2 = 2.55 (3)	*p* = 0.47	NA
1 episode	59.5%	62.5%			
2 episodes	5.4%	12.5%			
3 episodes	2.7%	0.0%			
COVID cases with COVID-related dyspnea	10.0%	4.0%	χ2 = 0.73	*p* = 0.39	OR = 2.67 (0.26, 27.38)

Abbreviations: COVID-19, coronavirus disease 2019; df, degrees of statistical freedom; FE, Fisher's exact test; MWU, nonparametric Mann–Whitney U test; NA, not applicable; OR, odds ratio.

In Table 4 , note that both a history of diabetes mellitus ( *p* < 0.001) and a history of any comorbidity ( *p* = 0.003) were associated with increased odds of having respiratory symptoms in the 55 patients who developed COVID. No other factors were predictors.

**Table 4:** Comparing coronavirus disease cases experiencing coronavirus disease-related respiratory dysfunction (other than cough or sore throat)

Characteristics	Nonrespiratory Sx	Respiratory Sx	Test statistic (df)	Significance, *p*	Odds ratio (95% CI)
*N*	51	4			
Age, mean	27.7	30.0	MWU = 127.0 (1)	*p* = 0.58	NA
Range	10–59	21–38			
% under 20 y old	21.6%	0.0%	χ2 =2.47 (2)	*p* = 0.29	NA
% 20–39 y old	60.8%	100.0%			
% 40 y and over	17.6%	0.0%			
% Female	13.7%	25.0%	χ2 = 0.38 (1)	*p* = 0.54	OR = 2.10 (0.19, 2.13)
Diabetes	2.0%	50.0%	FE	*p* = 0.049	OR = 50.0 (3.09, 810.5)
Hypertension (requiring treatment)	3.9%	0.0%	FE	*p* = 1.00	NA
Any currently active comorbid condition	5.9%	50.0%	FE	*p* = 0.037	OR = 16.0 (1.64, 156.6)
Years from surgery to pandemic, mean	3.86	3.00	MWU = 80.0 (1)	*p* = 0.60	
Range	1–15	1–6			
% under 5 y	70.6%	75.0%	χ2 = 0.25 (2)	*p* = 0.88	
% 5–9 y	23.5%	25.0%			
% ≥ 10 y	5.9%	0.0%			
When COVID diagnosed Before COVID vaccination	21.6%	0.0%	FE b	*p* = 0.53	NA a
After COVID vaccination	78.4%	100.0%			
COVID confirmed by nasal swab	90.2%	100.0%	χ2 = 1.47 (1)	*p* = 0.23	NA a
Number of episodes, mean	1.14	1.50	MWU = 140.0	*p* = 0.18	
Range	1–3	1–2			
1 episode	88.2%	50.0%	χ2 = 5.42 (2)	*p* = 0.066	NA
2 episodes	9.8%	50.0%			
3 episodes	2.0%	0.0%			

Abbreviations: df, degrees of statistical freedom; FE, Fisher's exact test; MWU, nonparametric Mann–Whitney U test; NA, not applicable; OR, odds ratio; Sx, symptoms.

a 0.48.

b 0.56.

## Discussion

As mentioned, one of the most significant potential drawbacks of using the phrenic nerve as an axon donor in severe BPI is the potential risk of respiratory impairment. This argument was heightened in the COVID era. Due to the severe lung disease that this pandemic produced in many patients with acute infections, the idea that a patient with a phrenic nerve transfer performed before the infection could have a respiratory problem was conceivable. Undoubtedly, the most important finding of this article is that none of the extensive series of patients analyzed herein ( *n* = 77) was admitted to the ICU or even to the hospital, intubated, or died in the acute setting of a COVID-19 infection ( Table 2 ). Of note, most patients ( *n* = 55 out of 77; 25 of 37 in Argentina vs. 30 out of 40 in the Taiwanese counterparts) underwent one or more than one acute COVID infection. The worst symptom that our patients showed was mild dyspnea, and this finding was not frequent ( *n* = 4, 7.3%). This percentage is similar to what similar populations—regarding age, sex, and clinical history—showed. 16 20 26 27 28 29 30 31 32

Indeed, the absence of complications in our population is biased by our patient selection process: to be selected for using a unilateral phrenic nerve transfer (end to end) for a brachial plexus reinnervation, the candidates have to be older than 3, not too elderly, have good respiratory function, and not obese. These patients typically did not exhibit intolerable complications during an acute COVID-19 infection. 22 33 34 35 36 However, the alleged selection bias is a protection circumstance accepted extensively in the centers where this type of nerve transfer is usually performed, and the findings demonstrate that this exclusion criterion worked well in our population.

When compared, the patients from the two countries involved didn't show statistically significant differences ( Table 3 ). Nevertheless, the rate of medical comorbidities (hypertension or diabetes, among others) was higher in Taiwanese patients than in Argentinians (12.5 vs. 2.7%, respectively). Patients from Argentina were younger (26.4 years old vs. 32.9 in Taiwan) and were more likely to be males (5.4% of females vs. 15% in Taiwan). Remarkably, two statistically significant geographical differences were found between the two countries: all patients in Taiwan were diagnosed with nasal swabs, a percentage that diminishes to 44% in Argentina, and most of the former group of patients had the first or subsequent COVID-19 acute episode after vaccination (60%), adversely to Argentinians (40%). The well-study phenomena of the COVID-19 pandemic hitting the isle of Taiwan later than in other continental countries (starting in 2021 vs. a rude start in 2020 in South and North America and in Europe) 37 38 39 40 41 can explain why most of the Taiwanese were already vaccinated when they experienced the first COVID-19 infection. The difference in access to nasal swabs in suspected cases between the two countries can not only explain the differences in diagnosis between the two groups but also the mildly higher tendency—yet not significant—verified in Taiwan to undergo more episodes of COVID-19.

When comparing COVID cases experiencing COVID-related respiratory dysfunction other than cough or sore throat ( Table 4 ), we found that diabetic patients, or those who showed any comorbidity at the moment of suffering an acute COVID-19 infection, evidence a higher percentage of respiratory symptoms (50 vs. less than 6%). Again, these data are unsurprising and align with what could be expected to happen in persons with a specific clinical report who are affected by a respiratory virus such as COVID-19.

The most significant limitation of statistical analysis was the small number of patients with respiratory symptoms, which markedly reduced the study's statistical power and eliminated the potential to perform regression analysis. Another limitation is the lack of a control group. However, indeed, it can be assumed that the risk of presenting an acute COVID-19 infection is similar in patients having a traumatic BPI when compared with another population matched accordingly in demographic terms.

## Conclusions

Although COVID was common in the studied patients, it was notable that the analyzed groups presented a very low rate of respiratory symptoms (just 4 of 77 patients) and lacked any need for hospitalization (all patients recovering at home), so no deaths were reported. Also, two predictors of increased odds of respiratory symptoms identified on bivariable analysis were a history of diabetes and a history of any severe medical comorbidity. It can be concluded that an acute COVID-19 infection was very well tolerated in our patients.
